# Evaluation of the Major Steps in the Conventional Protocol for the Alkaline Comet Assay

**DOI:** 10.3390/ijms20236072

**Published:** 2019-12-02

**Authors:** Mahsa Karbaschi, Yunhee Ji, Abdulhadi Mohammed S. Abdulwahed, Alhanoof Alohaly, Juan F. Bedoya, Shanna L. Burke, Thomas M. Boulos, Helen G. Tempest, Marcus S. Cooke

**Affiliations:** 1 Oxidative Stress Group, Department of Environmental Health Sciences, Florida International University, Miami, FL 33199, USA; yji008@fiu.edu (Y.J.); aabdu049@fiu.edu (A.M.S.A.); jbedo007@fiu.edu (J.F.B.); tboulos@fiu.edu (T.M.B.); mcooke@fiu.edu (M.S.C.); 2 Department of Human and Molecular Genetics, Florida International University, Miami, FL 33199, USA; 3 Department of Dietetics and Nutrition, Florida International University, Miami, FL 33199, USA; aaloh001@fiu.edu; 4 School of Social Work, Florida International University, Miami, FL 33199, USA; sburke@fiu.edu; 5 Biomolecular Sciences Institute, Florida International University, Miami, FL 33199, USA

**Keywords:** comet assay, DNA damage, DNA repair, oxidative stress, genotoxicity, human biomonitoring

## Abstract

Single cell gel electrophoresis, also known as the comet assay, has become a widespread DNA damage assessment tool due to its sensitivity, adaptability, low cost, ease of use, and reliability. Despite these benefits, this assay has shortcomings, such as long assay running time, the manipulation of multiple slides, individually, through numerous process steps, the challenge of working in a darkened environment, and reportedly considerable inter- and intra-laboratory variation. All researchers typically perform the comet assay based upon a common core approach; however, it appears that some steps in this core have little proven basis, and may exist, partly, out of convenience, or dogma. The aim of this study was to critically re-evaluate key steps in the comet assay, using our laboratory’s protocol as a model, firstly to understand the scientific basis for why certain steps in the protocol are performed in a particular manner, and secondly to simplify the assay, and decrease the cost and run time. Here, the shelf life of the lysis and neutralization buffers, the effect of temperature and incubation period during the lysis step, the necessity for drying the slides between the electrophoresis and staining step, and the need to perform the sample workup and electrophoresis steps under subdued light were all evaluated.

## 1. Introduction

Of all the methodologies for genotoxicity testing, single cell gel electrophoresis (the comet assay) has become one of the most popular DNA damage assessment tools by the virtue of its sensitivity, adaptability, economy, ease of use, reproducibility, and reliability [1]. The comet assay detects strand breaks and alkali-labile sites (which become strand breaks under alkali conditions) [2], arising from the interaction of various damaging intermediates (e.g., reactive oxygen species, e.g., hydroxyl radicals and hydrogen peroxide) with DNA [3], following exposure to a wide variety of genotoxins, such as ultraviolet radiation. This has led to its widespread application to in vitro and in vivo genotoxicity testing, e.g., nanomaterials [4], together with human biomonitoring [5], in environmental and occupational contexts [6], DNA repair studies, gene-specific assessments (using FISH-comet assay), and ecogenotoxicity (including the analysis of DNA damage in plants, and aquatic invertebrates and vertebrates) [7,8].

**Figure 1:**
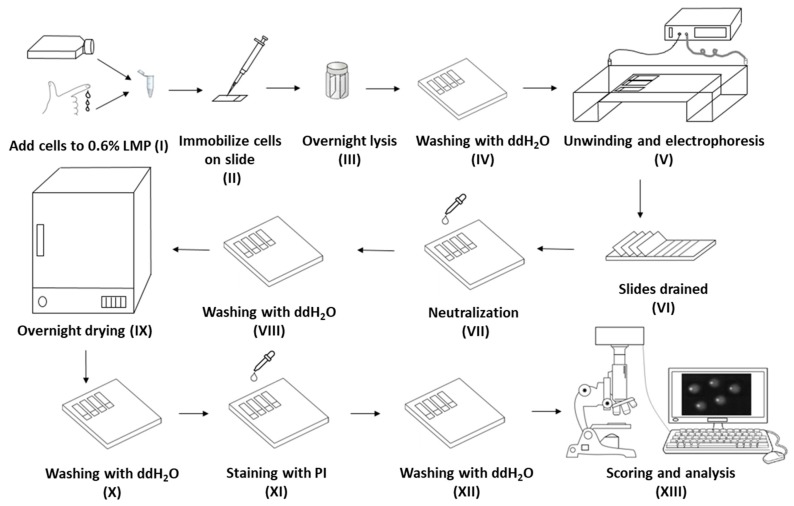
An overview of the alkaline comet assay procedure, reproduced from [10]. (**I**) A single cell suspension of the cells under investigation is mixed with low melting point agarose (LMP). (**II**) The cell/agarose mix is layered onto glass microscope slides, which are pre-coated with agarose, and the agarose allowed to cool and set. (**III**) The cells are lysed under high pH to leave only a nucleoid body (comprising nuclear DNA and some scaffold proteins), before (**IV**) washing with double distilled water (ddH_2_O). The presence of strand breaks, combined with high pH, allows the DNA to unwind. (**V**) Electrophoresis draws the fragmented DNA out of the nucleoid body, forming a “tail”. The amount of migration (the amount of DNA in the tail versus the head) is proportional to the initial amount of DNA damage. The slides are then (**VI**) drained, (**VII**) neutralized, and (**VIII**) washed with pure water, before (**IX**) drying overnight. Following further (**X**) washing in pure water, the slides are (**XI**) stained, (**XII**) washed, and finally (**XIII**) scored and analyzed, typically using fluorescent microscopy and image analysis software.

The principle of the comet assay is based on the migration of negatively charged, fragmented DNA strands when subjected to an electric field, facilitated by the presence of strand breaks. Briefly, a single cell suspension (following exposure to a physical or chemical agent, as relevant) in low melting point agarose is layered onto glass microscope slides and lysed in a high salt solution to generate nucleoid bodies. In the alkaline variant of the comet assay (alkaline comet assay, ACA), the DNA relaxes and unwinds, and is electrophoresed under alkali conditions, neutralized, stained with a fluorescent dye, and finally, visualized and analyzed under a microscope. Three key advantages of the comet assay are: (1) The need for relatively low numbers of cells [9], (2) applicability of the assay to all eukaryotic cells both in vivo and in vitro, and (3) the detection of the DNA damage at the level of individual cells. Despite these benefits, the assay possesses some shortcomings, such as (1) lengthy assay running time (up to three days); (2) numerous individual comet slide manipulations (see Figure 1 for an overview of the alkaline comet assay procedure), with risk of damage to the delicate agarose gels; (3) the challenge of working in a darkened environment to limit artefactual damage; (4) possible limited access of the exogenous DNA repair enzymes to all the adducts in the genome; and (5) reported inter- and intra-laboratory variation.

The development of different high-throughput platforms for the comet assay over the past six years has helped in minimizing the sample manipulations and handling time [10,11,12,13]. Moreover, the invention of a fully automated comet assay device for running the assay [14], or for the image analysis [15], promises to significantly decrease the assay run time and operator involvement, thus potentially improving the reproducibility of the assay. However, the comet assay is typically performed manually, using the widely reported conventional methods, and hence the assay remains time consuming and labor-intensive. Furthermore, some steps in the conventional protocol appear, to us, to have little rationale, and may exist, partly, out of convenience (e.g., overnight lysis), or dogma.

The aim of the present study was to critically re-evaluate our laboratory’s existing comet assay protocol, which is based upon those widely reported in the literature. The goals were to simplify, decrease cost and running time, whilst maintaining optimal conditions on a human dermal keratinocyte cell model. We also aimed to understand or provide evidence to justify why certain steps in the protocol are performed. Here, the shelf life of the lysis and neutralization buffers, the effect of temperature and incubation period during the lysis step, the necessity for drying the slides between the electrophoresis and staining step, and the need to perform the sample workup and electrophoresis steps in subdued (red) light were evaluated. The results following any changes to the experimental protocol were then compared with our laboratory’s existing historical positive and negative control database in terms of their consistency, as recommended by mammalian alkaline comet assay (ACA) protocols approved by the Organisation for Economic Co-operation and Development (OECD) [16].

Given its relative simplicity and widespread application, improving the comet assay protocol by solving the fundamental issues relating to experimental validation, and identifying the unclear factors that may affect the measurement of the DNA damage, may increase the great potential of this assay in clinical studies [17,18] and its other varied applications.

### Clinical Significance

DNA damage is key in the pathogenesis, and treatment, of many diseases, particularly cancer [19]. The comet assay repeatedly demonstrates a defective DNA damage response in cancer [9,20,21,22], leading to its proposed use in the clinical management of cancer [23]. It has been used to predict radiosensitivity in cervical cancer cells [24], and to predict bladder cancer chemosensitivity, radiosensitivity, and clinical outcome [9,25,26]. It has also been shown to be effective as a stand-alone, or an adjunct, test for detecting cancer, with a sensitivity and specificity comparable to cancer-specific tests [27]. On this basis, the comet assay must be robust and amenable to rapid, routine use.

## 2. Results

We evaluated factors associated with key steps in the alkaline comet assay process (see Figure 1): Lysis buffer shelf life (step III), neutralization buffer shelf life (step VII), lysis buffer temperature, incubation duration (step III), and airdrying of slides (step IX).

### 2.1. Evaluation of Lysis Buffer Shelf Life

**Figure 2:**
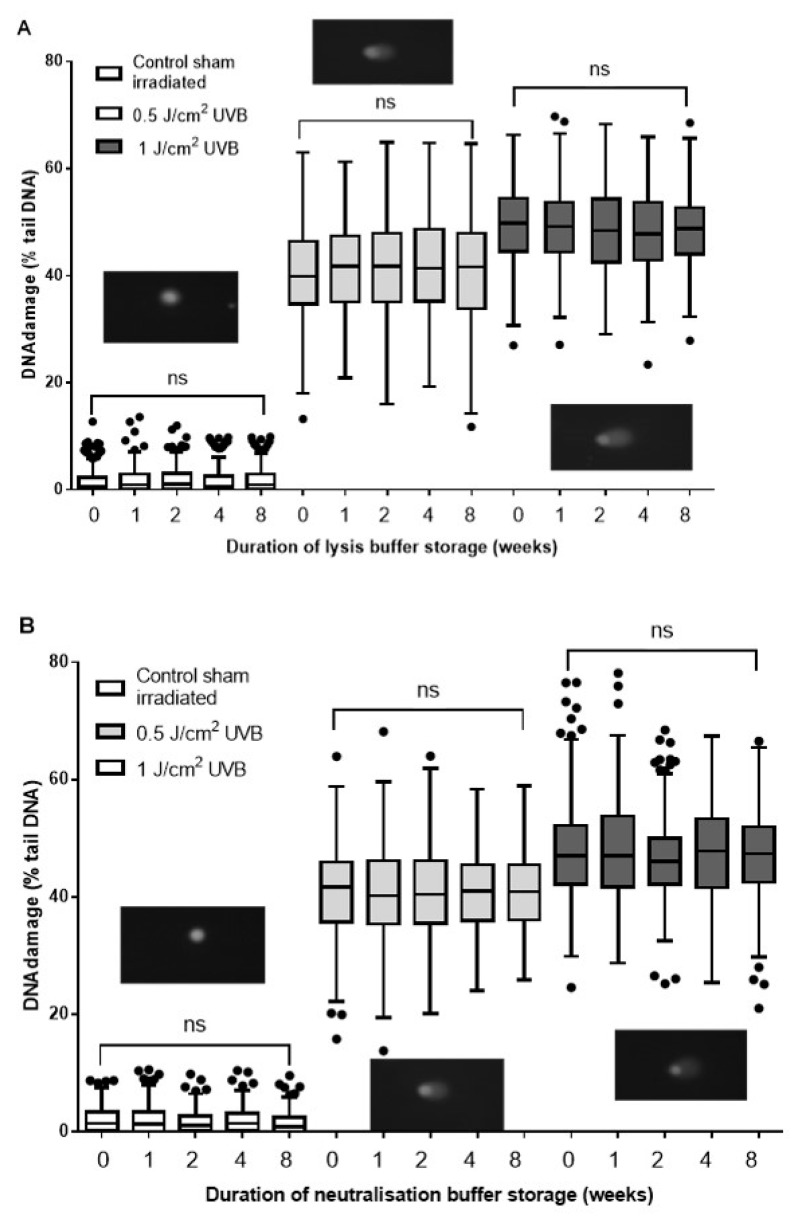
Evaluation of lysis buffer shelf life. Human keratinocytes (human adult low calcium high temperature cells, HaCaTs) were irradiated with ultraviolet B radiation (UVB), lysed, and electrophoresed following the standard protocol. The cells were then (**A**) lysed or (**B**) neutralized in neutralization buffer, each of which were made fresh on the day of the experiment (0 weeks), 1, 2, 4, or 8 weeks before the experiment, and stored at room temperature. Tukey box and whisker plot of median and inter-quartile range for results from two individual experiments, 100 determinations per treatment per experiment, and analyzed by Kruskal–Wallis test with Dunn’s multiple comparisons test. ns = not significant. Also shown are representative comet images of control and cells irradiated with 1 J/cm^2^ UVB (inset images).

In order to study the effect of lysis buffer effectiveness with age, we compared freshly made buffer with buffers made and stored for increasing periods of time. Figure 2A shows that the mean levels of background DNA damage did not differ significantly between the fresh and the oldest lysis buffer (1.6% and 1.94%, respectively; *p* > 0.05). Similarly, for the damaged samples, there were no significant differences in mean percentage tail DNA (%TD) values: 48.26% and 49.62% for 1 J/cm^2^, and 40.5% and 42.02% for 0.5 J/cm^2^, irrespective of whether they were lysed in fresh or any of the aged buffers.

### 2.2. Evaluation of Neutralization Buffer Shelf Life

In order to study the effect of neutralization buffer effectiveness with age, we compared freshly made buffer with buffers made and stored for increasing periods of time. The %TD for the slides neutralized with fresh neutralization buffer was consistent with our laboratory’s existing HaCaT dose response control database, used as a standard control in this experiment, and compared to the levels of damage in samples neutralized with the aged neutralization buffers (Figure 2B). The data obtained after scoring the comets indicated that there was no significant difference in %TD between the corresponding samples in each of the fresh or four “aged” neutralization buffers (*p* > 0.05). Also, visual comparison of the quality of the images from each group did not show any difference in terms of the level of background staining, debris, or shape of the comets.

#### 2.2.1. Evaluation of the Effect of Lysis Buffer Temperature, and Incubation Duration

**Figure 3:**
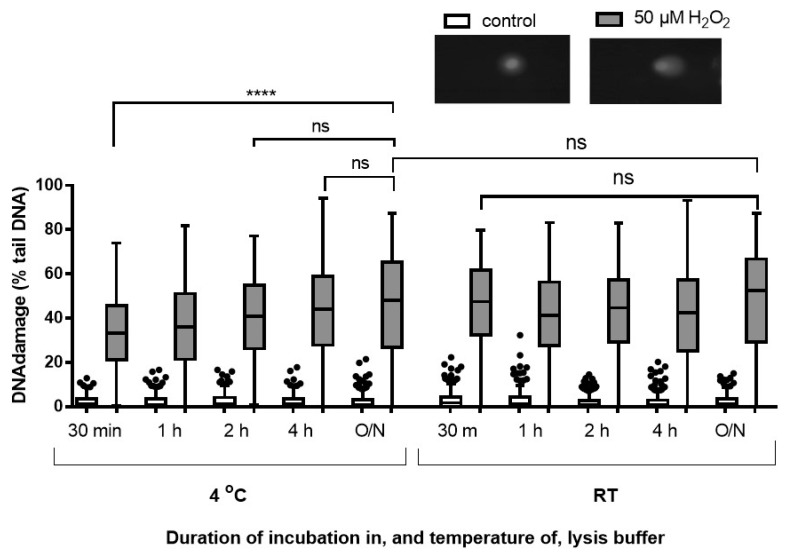
Effect of lysis duration and temperature. HaCaTs were treated with 50 µM H_2_O_2_. The slides were then incubated in lysis buffer for different durations of time at 4 °C or room temperature (RT). Tukey box and whisker plot of median and IQR of three independent experiments, 100 determinations per treatment per experiment. (O/N: Overnight; *p* > 0.05, Mann–Whitney test.) **** *p* < 0.0001, ns = not significant. Also shown are representative comet images of control and cells treated with 50 µM H_2_O_2_ (inset images).

In order to study the effect of lysis buffer temperature and duration of incubation, we compared our standard conditions (overnight at 4 °C) with shorter periods at 4 °C and room temperature. The results demonstrate that the level of damage detected in the samples lysed overnight at room temperature (42.15%) was higher than that in the samples under all other incubation periods at both room temperature and 4 °C (Figure 3). However, this was not significantly different from the level of damage in the cells treated for 30 min at room temperature (40.32%, *p* > 0.05), indicating that the ability of the comet assay to detect single strand breaks (SSB) and alkali-labile sites (ALS) does not change between 30 min and overnight incubation periods. This contrasts with the results obtained after 30 min versus overnight incubation at 4 °C (*p* < 0.0001 for H_2_O_2_-treated cells, and *p* = 0.01 for untreated cells), which emphasizes the key role of the length of incubation period when the samples are lysed at 4 °C (Figure 3). However, the data presented here show that the level of detected DNA damage after 4 h of incubation at 4 °C was not significantly different from that in the samples lysed overnight at 4 °C. These results demonstrated that the shortest required incubation period at 4 °C is 4 h, whereas the shortest required lysis period at room temperature was 30 min.

#### 2.2.2. Evaluation of Airdrying Slides for Different Times between Neutralization and Staining

**Figure 4:**
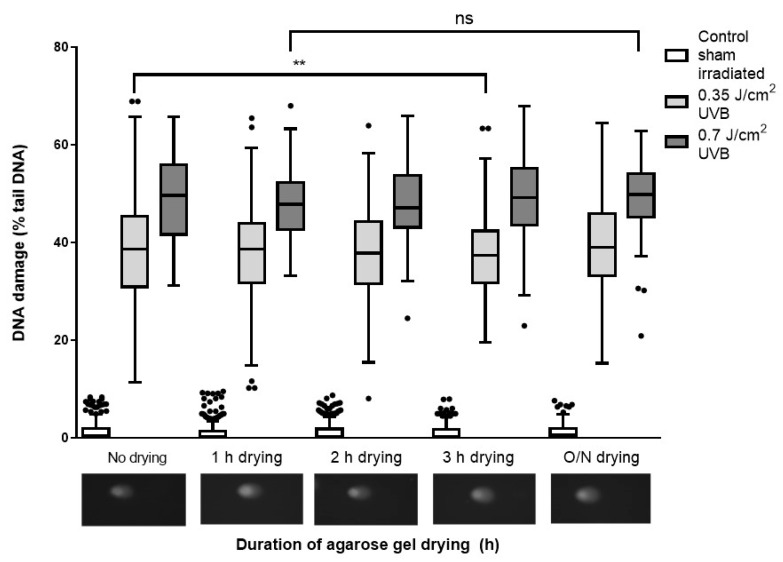
The influence of duration of dehydration of the slides prior to staining on the detection of DNA damage. HaCaTs were irradiated with UVB, and the alkaline comet assay (ACA) then performed until the end of neutralization step. At this point, and before the staining, two replicates from each treatment were dried for 1, 2, 3 h, and overnight, or not dried. Tukey box and whisker plot of median and IQR of two independent experiments, 100 determinations per treatment per experiment. (ns = not significant, ** represents *p* < 0.01, determined by the Mann–Whitney test.) Also shown are representative comet images of cells treated with 0.35 J/cm^2^ and dried for the indicated periods of time (inset images).

As seen in Figure 1, slides were washed, dried overnight, and then washed again, rehydrating the slides. The rationale for drying and then rehydrating did not seem clear, so we investigated the effect of this processing. For the slides that were dried then rehydrated prior to the staining, the level of DNA damage in the cells irradiated with 0.7 J/cm^2^ or sham irradiated were not significantly different within each group (Figure 4; *p* > 0.05). Also, for the cells damaged by 0.35 J/cm^2^ UVB, the difference between the cells dehydrated for 1, 2 h, or overnight was not significantly different from the non-dehydrated ones. However, the difference between the %TD on the slides dehydrated for 3 h and the ones which were not dried was significantly different (difference between the mean values was 2.48%; *p* < 0.01).

#### 2.2.3. Evaluation of Ambient vs. Subdued Illumination on the ACA

**Figure 5:**
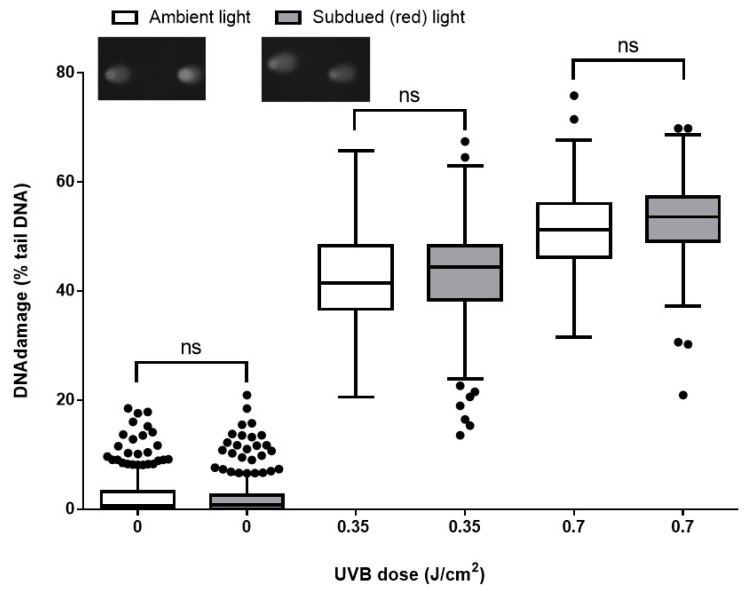
The effect of performing the comet assay under ambient vs. subdued light on DNA damage measurements. HaCaTs were either sham irradiated, or irradiated with 0.35 or 0.7 J/cm^2^ UVB. The alkaline comet assay was then performed in parallel in the dark room or under the laboratory white light. Tukey box and whisker plot of median and IQR of data from three independent experiments, 100 determinations per treatment per experiment. (ns = not significant, determined by the Mann–Whitney test.) Also shown are representative comet images of cells treated with 0.7 J/cm^2^ UVB and processed for the comet assay under ambient or subdued light (inset images).

The current recommendation is that all variants of the comet assay are performed under subdued light. As we hypothesized that the risk or artefactual formation of damage under ambient light would be low, we therefore tested this experimentally. The data obtained from the comet assay performed under white (ambient) or red (subdued) light were similar for the three tested doses of UVB on HaCaTs, and without any significant difference (*p* > 0.05; Figure 5).

## 3. Discussion

The ACA is a well-established, widely used method for evaluating DNA damage. However, some steps in the protocol appear to have become dogma, with little apparent evidence for their justification. In this report, we investigated some of these aspects to evaluate the scientific basis of their incorporation into the comet assay, aiming to make the assay quicker and easier.

The shelf life of buffer solutions in laboratories plays a major role in the accuracy and reproducibility of the analyses performed. According to the standard ACA protocol, the lysis and neutralization buffers should preferably be made fresh or be no more than two weeks old. A longer buffer shelf life could result in cost and time savings, through a reduction in preparation time and a decrease in reagent requirements. Our results demonstrated that both lysis and neutralization buffers were functionally stable for at least eight weeks at room temperature, even if the chemical composition/properties of the buffers altered with time. Further studies are required to determine the maximum shelf life of the buffers.

The percentage of low melting point agarose used, voltage strength, duration and temperature during electrophoresis, and the combined factors of temperature and duration of the lysis step all seem be critical roles in the reproducibility of comet assay [28,29]. At the lysis step, the operator can choose to pause and continue on the following day, or proceed with the next steps on the same day [30], although the longer duration of lysis incubation at 4 °C is most commonly performed [29]. Occasionally, simultaneous lysis of the cells and unwinding of the DNA in an alkaline solution is performed, which requires a longer incubation period [29,31]. According to the ACA protocol used in our laboratory, and many other laboratories [16,31], cells are lysed at 4–8 °C overnight (16–18 h), although others recommend 37–50 °C [31,32]. We did not test the effect of lysis at higher temperatures due to reports that this may induce artefactual damage [33]. Our data showed an increase in the detection of DNA damage in samples lysed overnight at 4 °C, compared to those lysed at 4 °C for 30 min. This is in agreement with Olive et al., highlighting that longer lysis at 5 °C allows for more DNA unwinding, and consequently increases the sensitivity of the assay [34]. However, our results demonstrated an equivalent sensitivity for HaCaT cells lysed for a minimum of 30 min at room temperature or overnight lysis at 4 °C, which saves a considerable amount of time. After a comparison between the effect of H_2_O_2_, Ro19–8022, and X-rays on different cell types, Enciso et al. showed that the effect of lysis duration depends on the damaging agent, and therefore the type(s) of DNA damage under examination [29,35]. OECD guidelines also emphasize the critical influence of lysis conditions for the conversion of alkali-labile lesions to strand breaks [16,35]. Furthermore, cell type-specific differences [36] may need to be taken into consideration when optimal lysis conditions are evaluated. It should be highlighted that during the lysis step, not only is the cellular membrane removed, but also the cytoplasm, nuclear envelope, scaffold, and histone proteins around the DNA [37]. Therefore, heterogeneity in nuclear chromatin structure between different cell types and its relationship with DNA-associated proteins may be a factor to consider for an efficient cell lysis (and subsequent steps). For example, it has been proposed that some cell types, such as keratinocytes, human spermatozoa, and buccal cells [38,39,40], may require proteinase K treatment to achieve a more substantial lysis [29]. Therefore, based on the cell type and the putative DNA damage under investigation, individual optimization of lysis conditions is recommended.

Another source of variation between different protocols is between neutralization and staining steps. In some protocols, the slides are stored for future scoring, with the gels dehydrated and fixed by immersing the slides in absolute ethanol or methanol [41]. Many ACA protocols, such as ours, specify airdrying of slides before the staining [16,42], with the intention of bringing all comets to a single plane [43] in the gel to eliminate the need to adjust the focal plane for each comet during scoring [11,44]. However, other protocols indicate staining slides immediately after neutralization [16]. Agarose is a long polymer of D and L galactose and when hydrated to form a gel, the carbohydrate makes helical fibers and creates channels through which the DNA strands migrate. During rehydration, the dried agarose gels can absorb up to 85% of their original water content [45], presumably returning to their original form, although this has not been reported explicitly. Therefore, the potentially significant role of drying and rehydration has been under consideration by us, as the rehydration could return the comets to different planes in the gel, if not fully rehydrated. Although many protocols recommend drying the slides between the neutralization and staining steps, our results clearly demonstrated that drying slides has an impact on the levels of damage, or the difficulty of scoring.

Lastly, we made a comparison between the results from the comet assay performed under ambient (white) vs. subdued (red) light conditions. It is always recommended to run ACA under subdued light conditions [31] although, to the best of our knowledge, this is the first report comparing the effect of ambient white light on the results of the ACA. Surprisingly, we noted a non-significant deference between the level of damage between the samples processed under ambient light compared to the ones which were processed under subdued lighting. However, the effect of performing the comet assay under ambient vs. subdued light following cell treatment with a different damaging agent, e.g., H_2_O_2_, Ro19-8022, should be tested, in case the type of damage present is a factor for whether or not artefactual damage is formed under ambient light.

## 4. Material and Methods

### 4.1. Lines and Culture Conditions

The human keratinocyte cell line, human adult low calcium high temperature (HaCaT), was used for all alkaline comet assay experiments in this study as a representative cell culture model system for normal cells [46].

### 4.2. Cell Treatments

For the treatment of HaCaTs with H_2_O_2_, cells were incubated with 50 μM H_2_O_2_ (in serum-free media) on ice for 30 min while covered with aluminum foil to prevent degradation of H_2_O_2_ due to light exposure. After 30 min, the media containing H_2_O_2_ were removed from the wells and the cells washed with PBS twice to remove any possible residual H_2_O_2_, and the cells harvested for processing in the comet assay. UVB irradiation was performed using a UVB bench lamp, and the intensity of the lamp was measured using a UVX Radiometer, in conjunction with a UVX-31 sensor (all of which were from Analytik Jena US LLC, Upland, CA, USA).

### 4.3. Alkaline Comet Assay (ACA)

After treatment, the cells were washed with PBS, and then analyzed by the high-throughput (HT) slide processing comet assay [10,47], incorporating our proposed modifications, as outlined below. Briefly, approximately 1.2 × 10^4^ cells, suspended in 80 μL of 0.6% *w/v* low melting point agarose (LMP) (Invitrogen, Carlsbad, CA, USA), were dispensed onto glass microscope slides pre-coated with 1% normal melting point agarose. The agarose was allowed to set under a 22 mm × 22 mm coverslip with the slides on a slide chilling plate (Cleaver Scientific, Rugby, UK). The cover slips were then removed, and the slides placed vertically in a High Throughput (HT) slide rack (Cleaver Scientific, Rugby, UK) and, from this point onwards, all the slides were processed in batches in the racks [10]. The slides were incubated at 4 °C (unless specified otherwise, see Section 4.4.3) in a lysis dish containing 200 mL of freshly made (unless specified otherwise, see Section 4.4.1) lysis buffer (2.5 M NaCl, 10 mM Tris–HCl, 100 mM disodium EDTA, pH 10, and 1% Triton X-100). The slides were then washed with water for 30 min prior to incubation in the HT electrophoresis tank containing alkaline electrophoresis buffer (300 mM NaOH, 1 mM disodium EDTA, pH ≥ 13, 4 °C) for 20 min. The cells underwent electrophoresis at 25 V (1.19 V/cm) for 20 min. The HT racks were removed from the tank and placed in a neutralization dish containing 200 mL of fresh (or aged, see Section 4.4.2) neutralization buffer (0.4 M Tris–base, pH 7.5) for 20 min, followed by washing with double distilled water. The slides were then dried overnight at 37 °C (unless specified otherwise, see Section 4.4.4). On the following day, the slides were rehydrated in water for 20 min and stained with 2.5 μg/mL propidium iodide solution (PI; Sigma, St. Louis, MO, US) for 20 min, followed by final washes. All procedures were performed under red light to decrease the potential formation of artefactual DNA damage (except in Section 4.4.5). Comets were visualized by fluorescence microscopy at 40× magnification, and Comet Assay IV software version 4.2 (Perceptive Instruments, Suffolk, UK). One hundred cells from duplicate slides were scored and the percentage of tail DNA (%TD) for each comet calculated. Each experiment was performed on at least two separate occasions.

### 4.4. Modified ACA Protocols

#### 4.4.1. Evaluation of Lysis Buffer Shelf Life

To evaluate the stability of the lysis buffer used in the comet assay, 1 L of lysis buffer was made fresh on the first day of running the comet assay, and also 1, 2, 4, and 8 weeks prior to the day of the experiment, and stored at room temperature (22–25 °C) inside a closed cabinet in a dark room with no windows. UVB-irradiated HaCaTs were embedded in LMP agarose and five identical groups of slides, each containing two gels per treatment were prepared. Each set of slides were then placed in a separate rack in a dish filled with one of the lysis buffers (freshly made, or up to 8 weeks old), overnight at 4 °C. The rest of the steps were performed according to Section 4.3. All the buffers, regardless of age, were prepared twice for testing in two separate experiments, on separate occasions.

#### 4.4.2. Evaluation of Neutralization Buffer Shelf Life

The stability of the neutralization buffer was tested as described above for the lysis buffer, with the shelf life of the neutralization buffer tested on buffers freshly made, or made 1, 2, 4, and 8 full weeks before the experiment, and stored at room temperature inside a dark cabinet. HaCaTs were irradiated with 0, 0.5, or 1 J/cm^2^ UVB, and embedded in LMP agarose on five identical sets of slides, two gels per treatment. All slides were then placed in one HT rack and the ACA performed up until the end of the electrophoresis step. At this point, each group of slides was transferred to one individual HT rack, and each rack placed in a separate neutralization dish, containing one of the five neutralization buffers, for 20 min. The slides were then returned to the HT rack and processed for staining. All the buffers, regardless of age, were prepared twice for testing in two separate experiments, performed on separate occasions.

#### 4.4.3. Evaluation of the Effect of Lysis Buffer Temperature and Incubation Duration

In order to test lysis buffer temperature and the duration, untreated or treated cells (50 μM H_2_O_2_, for 30 min, on ice) were embedded in LMP agarose. The slides were then incubated in lysis buffer at 4 °C or room temperature for a variety of time periods (0.5, 1, 2, 4 h, and overnight). Following lysis, all slides were transferred to one HT rack and processed for ACA as described (Section 4.3).

#### 4.4.4. Evaluation of Airdrying Slides for Different Times between Neutralization and Staining

HaCaTs were irradiated with 0.35 or 0.7 J/cm^2^ UVB on ice. ACA was then performed in one HT rack following the standard protocol described in Section 4.3, up to the end of the neutralization step. The slides were then removed from the HT rack and incubated for 0, 1, 2, 3 h, or an overnight at 37 °C to dry. At each time point, the slides were rehydrated and stained with 2.5 μg/mL PI solution.

#### 4.4.5. Evaluation of Ambient vs. Subdued Illumination on the ACA

HaCaTs were irradiated with 0.35 or 0.7 J/cm^2^ UVB and the ACA performed in parallel, both while the laboratory light was switched on (ambient light), or in the dark room under red light (subdued light), according to standard ACA protocols [48]. The room in which this test was run does not have windows, and the only light source was a ceiling Philips 32-Watt 4 ft linear T8 fluorescent white light tube. Potential UVA, UVB, and UVC emission from this light source was evaluated at bench level prior to running the experiment. None of the sensors detected any UV light at the level of the bench. The experiment was then performed following the ACA methodology outlined in Section 4.3.

### 4.5. Statistics

Statistical analysis of data was performed using GraphPad Prism software version 6.07 (San Diego, CA, USA), and the analysis of variance was tested by Kruskal–Wallis test with Dunn’s multiple comparisons test, and Mann–Whitney tests in which difference is regarded as significant if *p* < 0.05.

## 5. Conclusions

Here, we examined the effect of shelf life of the lysis and neutralization buffers, the effect of temperature and incubation period during the lysis step, the necessity for drying the slides between the electrophoresis and staining step, and the need to perform the sample workup and electrophoresis steps under subdued light on the results of the comet assay. We demonstrated that considerable further optimization of comet assay conditions was possible. It might be proposed that the remaining steps of the comet assay be revisited, and their necessity and optimization demonstrated, prior to incorporation into a standardized protocol. However, given the caveats from the literature that we noted earlier, e.g., optimization of lysis and other conditions for cell type- and damage-specific differences, as discussed further elsewhere [49], achieving a fully standardized “one-size-fits-all” protocol may not be possible.
